# Autophagy and the Bone Marrow Microenvironment: A Review of Protective Factors in the Development and Maintenance of Multiple Myeloma

**DOI:** 10.3389/fimmu.2022.889954

**Published:** 2022-05-19

**Authors:** Kamron R. Hamedi, Katrina A. Harmon, Richard L. Goodwin, Sergio Arce

**Affiliations:** ^1^ University of South Carolina School of Medicine Greenville, University of South Carolina, Greenville, SC, United States; ^2^ Research and Development Department, Organogenesis, Birmingham, AL, United States; ^3^ Biomedical Sciences, University of South Carolina School of Medicine Greenville, University of South Carolina, Greenville, SC, United States; ^4^ Prisma Health Cancer Institute, Prisma Health System, Greenville, SC, United States

**Keywords:** multiple myeloma, autophagy, bone marrow microenvironment, unfolded protein response, plasma cells, drug-resistance

## Abstract

The role of the unfolded protein response (UPR) in plasma cells (PC) and their malignant multiple myeloma (MM) counterparts is a well described area of research. The importance of autophagy in these cells, as well as the interplay between autophagy and the UPR system, has also been well studied. In this review, we will discuss the relationship between these two cellular responses and how they can be utilized in MM to account for the high levels of monoclonal immunoglobulin (Ig) protein synthesis that is characteristic of this disease. Interactions between MM cells and the bone marrow (BM) microenvironment and how MM cells utilize the UPR/autophagy pathway for their survival. These interacting pathways form the foundation for the mechanism of action for bortezomib, a proteasome inhibitor used to modify the progression of MM, and the eventual drug resistance that MM cells develop. One important resistance pathway implicated in MM progression is caspase 10 which attenuates autophagy to maintain its prosurvival function and avoid cell death. We lay a groundwork for future research including 3D *in vitro* models for better disease monitoring and personalized treatment. We also highlight pathways involved in MM cell survival and drug resistance that could be used as new targets for effective treatment.

## Introduction

Cellular stress activates several processes which either restore cellular homeostasis or commit the cell to cell death. These processes include the UPR, autophagy, hypoxia, and mitochondrial function, which are part of the global endoplasmic reticulum (ER) stress response.

The UPR plays a crucial role in restoring homeostasis following accumulation of unfolded/misfolded proteins. This cellular response is regulated by three signaling mechanisms: inositol-requiring enzyme 1 (IRE1), PKR-like endoplasmic reticulum kinase (PERK), and activating transcription factor 6 (ATF6). All three activate specialized transcriptional programs mediated by distinct transducers: spliced x-box binding protein 1 (XBP-1) for IRE1, activating transcription factor 4 (ATF4) for PERK, and cleaved-ATF6 for ATF6. The UPR specifically is triggered by cellular stress arising from altered environmental conditions or intracellular changes.

Autophagy is a cellular mechanism of self-clearance by which undesired intracellular components are degraded. Autophagy removes misfolded or aggregated proteins, clears damaged organelles, and eliminates intracellular pathogens. There are three types of autophagy within most cells: chaperone-mediated autophagy (CMA), micro-autophagy, and macro-autophagy (commonly referred to as “autophagy”).

In CMA, targeted proteins are translocated across the lysosomal membrane in complex with chaperone proteins, which are recognized by the lysosomal membrane receptor lysosomal-associated membrane protein 2A (LAMP-2A), resulting in protein unfolding and degradation. Micro-autophagy is a nonselective lysosomal degradation pathway that targets damaged cellular components. Micro-autophagy produces vesicles formed by lysosomal membrane invagination transferring cellular components into the lysosomal lumen to induce the degradation of its cytosolic constituents. Macro-autophagy (autophagy) delivers cytoplasmic cargo to the lysosome through intermediary double membrane-bound vesicles known as autophagosomes, which fuse with the lysosome to form an autolysosome. Both micro-autophagy and autophagy are capable of engulfing large cellular structures. After degradation, autophagy products from all three pathways are released into the cytosol to generate energy stores for the cell such as ATP generation.

Activation of the UPR can trigger changes in autophagy which in turn can modulate the UPR, exemplifying crosstalk between these processes. Given these potential interactions, UPR and autophagy dysfunction has been implicated in several human diseases including diabetes, neurodegeneration, and cancer. A better understanding of the complex interactions between the UPR and autophagy could lead to novel therapeutic approaches to these and other related pathological conditions.

This review will focus on the cross talk between the UPR and autophagy as a survival mechanism for MM cells, its connection to bortezomib resistance, and the role of the BM microenvironment. A critical connection between the UPR and autophagy is XBP-1. XBP-1 drives B-cell maturation into PCs and MM cells, and promotes autophagy through downstream expression of ATG5. Nuclear factor kappa beta (NFkB) is an important mediator of many signaling pathways including XBP-1 and is often constitutively activated in MM cells explaining why the UPR and autophagy are upregulated in this disease process. These pathways are further enhanced in the BM microenvironment as NFkB activity is increased when the integrin receptor VLA-4 on MM cells bind either VCAM-1 on bone marrow stromal cells (BMSCs) or fibronectin in the extracellular matrix (ECM). Cytokines released by BMSCs such as IL-6 and APRIL also increase NFkB and autophagy activity. Bortezomib, a 26S proteasome inhibitor, causes both increased proteasome stress and decreased NFkB activity in MM cells leading to cell death in BMSCs. However, this cell death eventually selects for BMSCs that produce transforming growth factor beta (TGF-β) in response to bortezomib. TGF-β increases pathways leading to prosurvival autophagy and production of IL-6 and other cytokines by stromal cells. These cytokines also promote prosurvival autophagy in MM cells causing resistance to the effects of bortezomib. Recent research has recognized the importance of these pathways and used 3D models to better study MM development and survival *in vitro*. Future research should utilize patient specific 3D samples to monitor disease progress, circumvent treatment resistance, and search for new therapeutic targets specific to the patient’s type of MM.

## The Unfolded Protein Response in Plasma Cells

**Figure 1:**
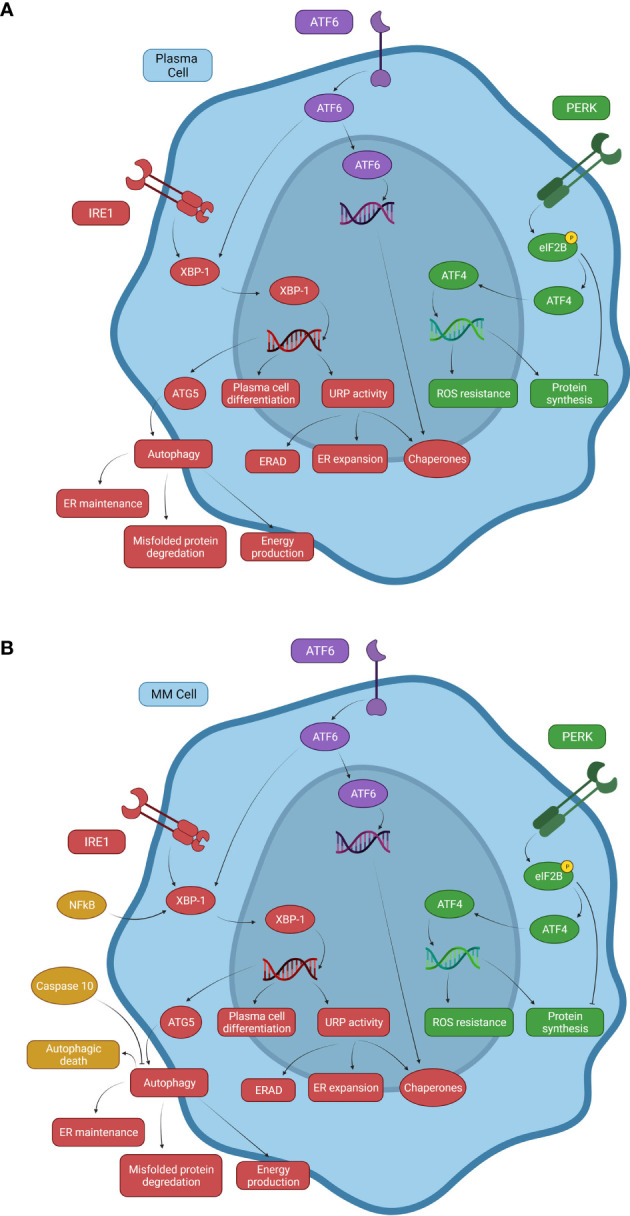
Signaling mechanisms of the UPR system and autophagy in normal and malignant plasma cells. **(A)** Normal B cells and PCs use three primary pathways associated with the UPR that respond to proteasome stress. The PERK pathway inhibits eIF2B through phosphorylation to promote ATF4 expression, inducing transcription of ROS resistance genes. PERK causes both decreased and increased protein synthesis, but overall decreases protein synthesis. The ATF6 pathway can drive chaperone protein gene expression and XBP-1 activity in the IRE1 pathway. The IRE1 pathway increases XBP-1 activity, inducing expression of ATG5, an important factor involved in autophagy activation, linking the UPR with autophagy. **(B)** A representation of some of the changes to these systems in multiple myeloma. NFkB is constitutively activated in a significant proportion of MM neoplasms due to activating mutations. Elevated NFkB activity induces XBP-1 expression, promoting autophagy and uncontrolled B cell transformation to MM cells. Caspase-10 expression serves as a survival factor for MM cells by attenuating autophagy and preventing autophagic death due to cellular overactivation.

The UPR utilizes three cellular processes to recover homeostasis: 1) the PERK pathway attenuates the load of nascent protein in the ER through global suppression of protein translation; 2) ATF6 and IRE1 together improve the protein folding capacity of the ER *via* upregulation of chaperones and foldases; and 3) IRE1 signaling facilitates ER associated degradation (ERAD) of misfolded proteins due to regulated IRE1-dependent mRNA decay (RIDD) (**Figure 1A**) (1). Under normal conditions, the three transmembrane proteins (PERK, ATF6, and IRE1) are maintained in an inactive state by their association with glucose regulated protein 78 (GRP78). During the UPR, increasing unfolded protein levels in the ER lumen bind to GRP78, resulting in dissociation of PERK, ATF6, and IRE1 from GRP78, initiating a signal transduction cascade.

IRE1 exerts a pro-survival role by inducing the splicing of two mRNA stem-loops and activation of the X-box-binding protein -1 (XBP-1) and RIDD, which then acts to reduce ER proteotoxic stress by inhibiting protein synthesis *via* mRNA template elimination (**Figure 1A**) (2, 3). XBP-1 modifies the folding capacity of the ER, decreasing proteosome stress, and increasing autophagic activity by upregulating expression of downstream UPR and autophagy related (ATG) genes (4). Activated XBP-1 enhances DNA translation of XBP-1 itself as well as PERK, ATF4, and eukaryotic translation initiation factor 2 alpha kinase 3 (eIF2ak3) which are all important to UPR activity (5). XBP-1 also binds the promoter of the immunoglobulin heavy chain-binding protein (BiP) gene, an important chaperone that both aids in the UPR and is a sensor for IRE1 activation itself. As BiP increases, more chaperones are available to eliminate improperly folded proteins and free IRE1 is bound to decrease UPR activation (6, 7). eIF2ak3 has been shown to upregulate ATG5 and beclin-1, proteins important to the activation of autophagy (8, 9). These pathways connect XBP-1 activity with not just the UPR but autophagy activation as well. Additionally, when ATF6 is activated and proteolytically cleaved, it becomes a transcription factor for many downstream UPR genes including XBP-1 (4, 10). This cooperation between the IRE1 and ATF6 pathways leads to a marked increase in XBP-1-mediated reduction of ER proteosome stress (11).

PERK activation leads to phosphorylation and resulting inactivation of eukaryotic translation initiation factor 2B (eIF2B). The inactivation of the eIF2B complex prevents ribosomes from recognizing inhibitory sequences in the untranslated regions of ATF4, a transcription factor that increases expression of anti-redox-genes and protein synthesis (1, 11, 12). Under normal cellular conditions, eIF2B aids in the function of the upstream open reading frame 1 (uORF1) to cause scanning ribosomes to bind and translate the upstream open reading frame 2 (uORF2) of ATF4 mRNA. uORF2 is inhibitory to ATF4 expression and silences this pathway under normal conditions. Phosphorylated eIF2B disrupts uORF1 function allowing ribosomes to translate ATF4 and contribute to the UPR (**Figure 1A**) (13). This results in a decrease in protein synthesis, which in conjunction with IRE1 and ERAD work to reduce unfolded protein build-up (1). PERK also activates NRF2 (Nuclear factor erythroid 2–related factor 2) *via* phosphorylation, which participates in the regulation of oxidative stress (4). This illustrates a possible dual role of PERK in attenuating the UPR as increased oxidative stress, which can be caused by unfolded proteins, triggering activation of the UPR (4). By increasing NRF2 activity to combat rising oxidative stress, PERK can alleviate one of the inciting factors of the UPR while simultaneously activating the UPR *via* ATF4 upregulation to help mitigate cellular stress.

B-cell differentiation into PCs is partially regulated by the IRE1 pathway through expression of XBP-1 (12, 14). Expression of XBP-1 is enhanced by the binding of B cell-activating factor (BAFF) and a proliferation-inducing ligand (APRIL) to specific transmembrane receptors (15). BAFF and APRIL have multiple functions in B cell and PC survival and differentiation when binding receptors such as BCMA (B-cell maturation antigen) and TACI (transmembrane activator and CAML interactor). When BAFF binds TACI, the TACI receptor recruits tumor necrosis factor receptor-associated factors (TRAF) and p65 which are associated with enhanced NFkB expression (16). NFkB has been shown to be an important regulator of XBP-1 expression in breast cancer cell lines, linking NFkB activity with the XBP-1 pathway (17). The binding of BAFF and APRIL to the TACI receptor initiates signaling pathways leading to XBP-1 expression and PC differentiation as well as other pro-survival effects (16, 18). XPB-1 deficient mice (XBP-1^-/-^) have reduced numbers of BM PCs compared to wild type mice (19). A similar study using Recombination activating 2 (Rag2)-XBP-1 deficient mice demonstrated decreased Ig production despite normal B-cell proliferation and germinal center formation. Conversely Rag2/XBP-1 double KO mice that had XBP-1 activity restored produced significantly more Ig than Rag2/XBP-1 deficient control mice suggesting that XBP-1 and the UPR contributes to PC maturation, though maturation is driven by a multitude of other factors (14, 20). Increased XBP-1 expression has been noted in MM cells, however no mutation contributing to initial pathogenesis has been identified in its genes (21). However, mutations in XBP-1 that decrease expression have been associated with bortezomib resistance during MM treatment (22). These findings highlight the importance of the IRE1-XBP-1 axis in the pathogenesis of MM, though this is likely an effect of upstream mutations elsewhere in the signaling pathways.

## Autophagy in Plasma Cells

Autophagy is a catabolic process that delivers proteins, cytoplasmic components, and organelles to lysosomes for degradation and recycling. ATG genes regulating autophagy can be activated by nutrient starvation through inhibition of target of rapamycin (mTOR) signaling or by the UPR as aggregated unfolded/misfolded proteins accumulate in the ER. Initially autophagy was thought to be a dispensable mechanism for PC homeostasis, but recent studies have demonstrated that autophagy plays a role in PC survival and differentiation (11, 23, 24). ATG5-deficient mice have an increased PC death rate resulting in decreased Ig production (23). Interestingly, the inhibition of autophagy led to increased Ig production, as well as ER stress and PC death (23). Autophagy may negatively regulate Ig production while promoting PC survival so that Ig production over the lifetime of the PC is increased (23, 24). Autophagy also plays a role in the homing of memory PCs to the BM (23, 25). When ATG5 deficient mice were examined for memory PCs, it was found that the BM contained almost exclusively PCs with residual ATG5 expression and active autophagy (23). Given that short-lived PC generation in these mice still occurred outside the BM, this result pointed to the role of autophagy in memory PC selection for homing to the BM, rather than PC differentiation from precursor B-cells (23, 25).

While autophagy contributes to the formation of BM-associated memory PCs, it also promotes survival by increasing energy production (23). Nearly twice as many activated B-cells died in ATG5 deficient mice compared to their littermate controls. The mechanism for starvation induced autophagy is mediated by mTORC1, which usually acts as an autophagy inhibitor (26). Starvation temporarily inhibits mTORC1 *via* the AMPK pathway, allowing increased autophagy for the breakdown of cellular components into basic metabolites that are shuttled into various energy production pathways (27). If adequate energy production is restored, mTORC1 activity is reestablished and autophagy is inhibited (27).

Autophagy acts in synergy with the ubiquitin/proteasome system. As proteasome stress increases, p62/SQSTM1 (sequestosome 1) forms aggregates with ubiquitin-tagged proteins that have not yet been degraded (28). This aggregation aids protease recruitment for protein degradation *via* the ubiquitin proteosome system (UPS) (29). However, p62 can also initiate selective autophagy (28). This dual role of p62 allows autophagy to complement UPS activity and promote cell survival. Of interest, both increased and decreased p62 has been found to increase autophagy through different pathways. It is hypothesized that this is due to its role as a regulatory of autophagy (30).

Autophagy may also help reduce proteasome stress by inducing ER degradation. XBP-1-induced PC differentiation is characterized by expansion of the ER membrane as a normal function of the UPR to increase the capacity of the ER to handle unfolded proteins (23). However, autophagy is associated with degradation of the ER since ATG5 deficient B cells showed significantly expanded ER compared to their normal counterparts. It has been suggested that this degradation may provide phospholipids for increased autophagy and eliminate misfolded proteins as they become trapped in newly formed phagosomes (23, 24).

## The UPR, Autophagy, and Multiple Myeloma

In cancer cells, metabolic stress induced autophagy is a cellular alternative source of energy and metabolites, enhancing adaptive cell responses to cancer therapies. Specifically, in hematological malignancies, autophagy plays an essential role attenuating drug-induced cell death *via* chemoresistance. MM is a heterogenous, hematological malignancy characterized by the expansion of monoclonal PCs (MM cells) in the BM. MM cells produce large amounts of monoclonal Ig resulting in a potential build-up of abnormally folded Ig molecules in the ER. Due to their high proliferative rate and Ig synthesis capacity, MM cells often accumulate toxic protein aggregates and therefore strongly depend on the UPR and autophagy for survival.

Several mutations are associated with MM and its progression. These mutations frequently comprise translocations of Ig gene enhancers causing overexpression of a variety of cellular proto-oncogenes (31). Other mutations in MM cells result in increased survival and proliferation. Activating mutations of NFκB, a transcription factor known for its anti-apoptotic activities, increases survival of MM cells (**Figure 1B**) (32–34). BAFF is also produced by malignant B-cells like MM cells and acts as an autocrine factor on MM cell BCMA and TACI receptors (35). As previously discussed, activated TACI receptors increase NFkB expression (16). NFκB is critical for B-cell differentiation through upregulation of Blimp-1 and XBP-1 expression, linking NFκB mutations to increased PC differentiation, function, and survival. This is consistent with observations that increased XBP-1 levels are characteristic of some forms of MM (21). Since PC activity reliant on UPR upregulation causes increased Ig production, mutations that upregulate the UPR and increase MM cell survival may account for the accumulation of malignant PCs and hypergammaglobulinemia observed in MM (25).

**Figure 2:**
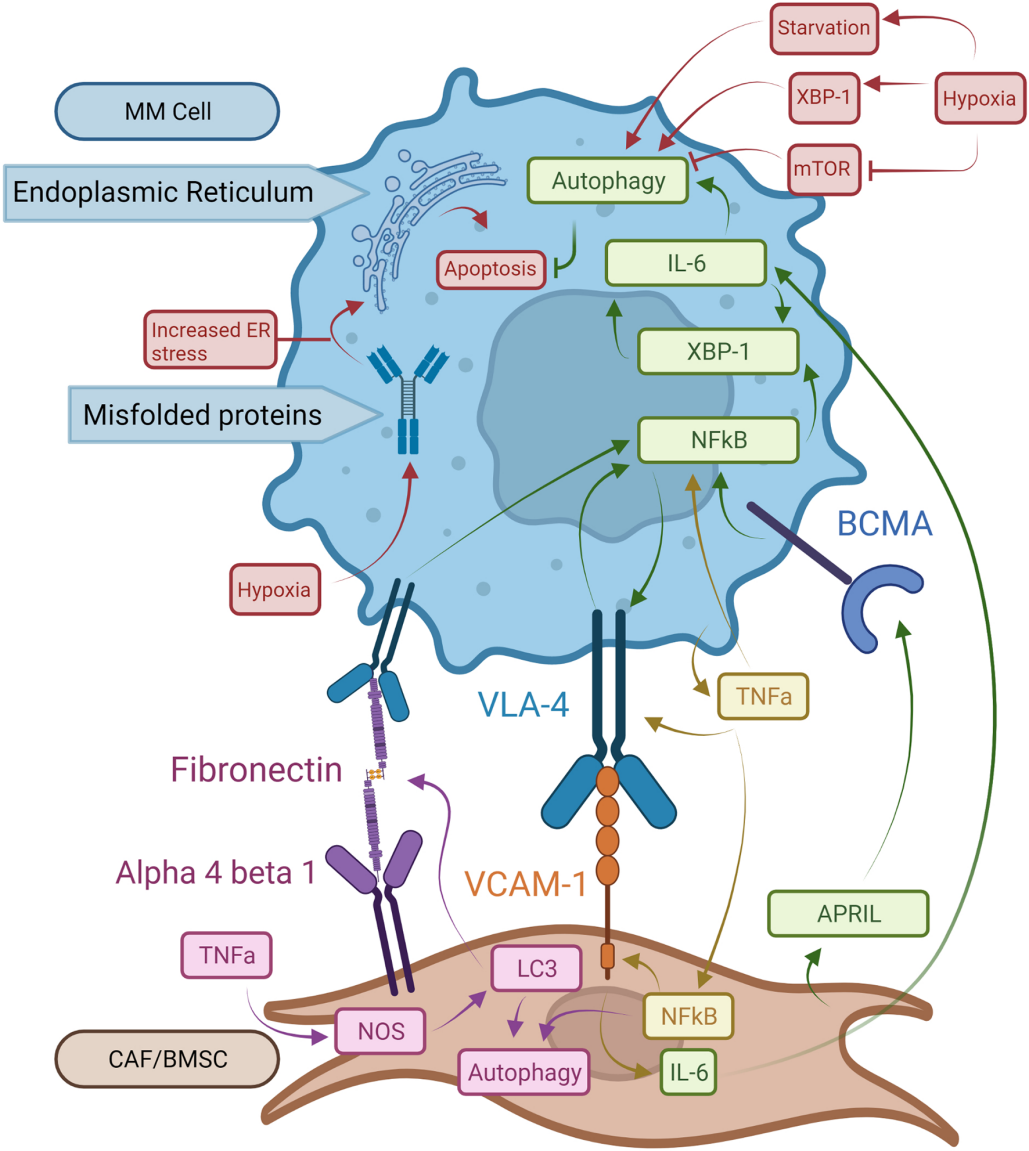
Pro-oncogenic signaling mechanisms in MM cells and BM microenvironment structures. BCMA receptors activated by APRIL increase NFκB activity. This leads to XBP-1 upregulation with enhanced autophagy and expression of VLA-4 on MM cells. Binding of VLA-4 with VCAM-1 on BMSCs induces NFkB expression in MM cells resulting in a stimulatory feedback loop. Bound VCAM-1 in turn increases IL-6 synthesis in BMSCs which promotes autophagy and survival in MM cells. TNF-a made by MM cells increases NFkB expression in both MM cells and BMSCs and augments VLA-4 expression on MM cell surfaces. NFkB in BMSCs boosts expression of VCAM-1 enhancing binding to MM cells. In BMSCs, TNF-α induces autophagy via LC3, which also binds fibronectin mRNA increasing fibronectin expression in BMSCs. Extracellular fibronectin protein can bind to VLA-4 on MM cells and enhance NFkB activity. Hypoxia exacerbates the UPR on the ER by driving autophagy via XBP-1 expression. Finally, mTOR works as a suppressor of autophagy, which is inhibited by hypoxia.

The NFκB pathway increases autophagy *via* binding a promoter sequence in the Beclin-1 gene inducing Beclin-1 gene expression (36, 37). Beclin-1, along with microtubule-associated protein light chain 3 (LC3), regulates autophagy *via* initial nucleation of the isolated membrane and the elongation stages. LC3 also functions in autophagy substrate selection and autophagosome biogenesis (**Figure 2**) (9, 38, 39). Increased Beclin-1 and LC3 expression has been noted in MM cells and is associated with favorable outcomes with median overall survival being 1,171 and 934 days, respectively (40). Additionally, high autophagy-related marker expression was associated with a favorable prognosis in a variety of malignancies such as non-small-cell lung carcinoma and some non-Hodgkin lymphomas (40). MM is caused by a variety of mutations leading to multiple disease subtypes. While NFkB activity is important in many forms of MM, those that are particularly reliant on autophagy may be more susceptible to available treatments or may be more easily be pushed to autophagic cell death (41). The mechanism by which high expression levels of autophagic markers produced better outcomes is not yet fully understood and would require more research in order to identify. Autophagy has both pro- and anti-tumorigenic effects depending on the cancer-specific process and microenvironment present (40). Therefore, the prognostic and pathophysiological implication of autophagic markers should be investigated in different cancers individually, including MM which is itself a highly heterogeneous cancer.

Autophagy may play a role in the progression of monoclonal gammopathy of undetermined significance (MGUS) to full MM. MGUS is defined as an early, non-cancerous stage of MM characterized by low M-protein levels, <10% clonal PCs, and lack of myeloma-defining symptoms (42). A recent study compared the BM of MGUS and MM patients. While the PCs in both samples were incredibly variable with many shared characteristics, only the MM patient PCs showed significantly increased autophagy (43). This finding highlights the potential importance of autophagy for the progression of MGUS to MM. Very little research has focused on the role of autophagy in the development of MGUS and smoldering MM, other stage of MM pathogenesis, into full MM. One study reported increased heat shock protein family A member 5 (HSPA5), a gene important for GRP78-induced autophagy, expression in MM PCs compared to MGUS PCs (44). Future research is needed to better define the role of autophagy in MGUS and smoldering MM and describe the role it has in disease progression.

Studies in PERK knockout mice that develop a MM-like disease associated with reduced UPR activity showed increased levels of MM cell death. In both this mouse MM model as well as in patients treated with bortezomib, cell death was mediated by autophagy instead of apoptosis (45, 46). Additionally, the excessive activation of autophagy triggered by Beclin-1 in other studies led to autophagic cell death in MM cells in those studies (47, 48). This suggests that treatment of MM using proteasome inhibitors, particularly those inhibiting the UPR, could cause increased autophagy in response to increased proteotoxicity from diminished UPR activity (46, 47). But as this autophagic response increases, the prosurvival effects of autophagy against proteotoxicity can be insurmountable and trigger autophagic cell death. However, MM cells can counteract autophagic cell death through interferon regulatory factor 4 (IRF4)-induced caspase 10 upregulation (**Figure 1B**) (24, 41, 46). When caspase 10 is active, it binds and degrades Bcl-2-associated transcription factor 1 (BCLAF1), which would otherwise bind and inactivate the anti-apoptotic Bcl-2 (B-cell lymphoma 2) gene. With BCLAF1 limited, Bcl-2 is free to bind Beclin-1 and inhibit autophagy (41, 46). However, if caspase 10 is inhibited, increased levels of autophagic death is observed due to increased BCLAF1 and Beclin-1 and decreased Bcl-2 activity, establishing the role of caspase 10 in MM cell survival (46).

## The Bone Marrow Microenvironment and Autophagy

The BM microenvironment consists of cellular and non-cellular compartments which are responsible for supporting the survival of PCs and MM cells. Numerous factors have been described which contribute to memory PC and myeloma survival (49, 50). Additionally, PCs also require specific cell-cell interactions with BMSCs to survive by inducing stromal cell production of interleukin 6 (IL-6) and other factors which enhance PC survival (49, 51, 52).

In MM patients, IL-6 has been identified as a key factor in pathogenesis by inhibition of apoptosis in MM cells (53, 54). IL-6 also interacts with several factors involved in the development and progression of MM, such as adhesion molecules, tumor suppressor genes, and oncogenes (55). Clinically, many MM patients have elevated serum levels of IL-6, which is associated with poor prognosis (56). While IL-6 production is one of the downstream effects of XBP-1 activity for PC survival, IL-6 itself can also induce XBP-1 upregulation (55, 57, 58). This cyclical pathway between IL-6 and XBP-1 production could explain increased IL-6 levels observed in MM patients (**Figure 2**). As IL-6 is induced by XBP-1 and the BM environment, increased levels of IL-6 induce additional XBP-1 production leading to increased UPR and autophagy activity and therefore greater Ig production (23). APRIL and BCMA secreted by BM stromal cells play critical roles in maintaining the growth and expansion of PCs within the BM *via* XBP-1 as well (**Figure 2**). When APRIL binds to BCMA, NFkB-mediated XBP-1 activation of UPR and autophagy pathways in PCs promote signaling pathways mediating cell growth and survival (59, 60). It was also shown in recent 3D models that MM and MGUS BM core samples exposed to IL-6 had increased CD138+ PCs after five days, indicating that in both stages of disease IL-6 provides significant pro-survival activity (43). This, in addition to the previous pathways for disease progression, demonstrates how important IL-6 is to MM pathogenesis and maintenance.

MM cell localization within the BM allows for cell-cell interactions between tumor and non-tumor BM cells. Interactions between PCs and the ECM within local BM niches contribute to MM cell survival and therapeutic resistance (61). BM microenvironments are comprised of BMSCs and ECM proteins including fibronectin, which is highly expressed throughout the BM (62). TNF-α produced by the BMSCs is a key mediator of adhesion molecule expression including ICAM-1, LFA-1, and VLA-4. This is accomplished through stimulation of NFκB signaling pathways in both BMSCs and homed MM cells (**Figure 2**) (63). Constitutively active NFkB pathways in MM cells significantly increase adhesion molecule expression and increase binding to BMSCs, further increasing NFκB activity in both cell types (63). The binding of VLA-4 to fibronectin also triggers NFκB signaling and is associated with cell adhesion-mediated drug resistance (**Figure 2**) (59). Fibronectin in the BM is largely produced by BMSCs (64). TNF-α has been shown to increase fibronectin production in coronary artery smooth muscle cells through the production of nitric oxide (65). Nitrous oxide (NOS) was shown in ductus arteriosus tissue to increase fibronectin production when LC3 bound fibronectin mRNA to enhance the efficiency of fibronectin translation (66). Thus, overexpression of LC3 due to NFkB upregulation may cause accumulation of fibronectin, which in turn triggers NFκB activation and autophagy in MM (**Figure 2**). These pathways could help explain recent findings that MM patients have increased fibronectin in the BM stroma compared to MGUS patients. This increase in fibronectin was accompanied by an increase in CD138+ cells, which is a marker for PCs (43). These pathways could help explain both findings, suggesting that increased fibronectin is integral to the development of MM and the increase in PCs associated with the disease, though further research would be needed to confirm these observations.

Another factor important to MM progression in the BM is hypoxia, which stimulates UPR and autophagy pathways. Hypoxia, which is present in MM microenvironments, causes cell stress from unfolded proteins. This is due to both a decrease in oxygen-dependent protein folding leading to UPR activity. Hypoxia also causes starvation from the lack of oxidative phosphorylation, promoting autophagy (67–69). Many proteins, such as low-density lipoprotein receptors and various mitochondrial proteins, rely on oxygen for proper folding and cause significant cellular stress under hypoxic conditions (70, 71). Hypoxia-driven UPR acts *via* the XBP-1, PERK, eIF2a, and ATF4 pathways (67, 72–74). XBP-1 interacts with the cell’s response to hypoxia by upregulating hypoxia-inducible factor 1-alpha (HIF1a) expression and the expression of HIF1a targets like vascular endothelial growth factor (VEGF) (67, 74). Furthermore, hypoxia is known to upregulate autophagy, possibly through upregulation of XBP-1 and in response to ER stress (**Figure 2**) (24, 25, 75). Hypoxia has also been shown to interact directly with autophagy activation through inhibition of mTORC1, which inhibits autophagy (**Figure 2**) (75).

## Drug Resistance in Multiple Myeloma

Activation of pro-survival autophagy can be induced in response to multiple stressors such as oxygen/nutrient depletion, ECM degradation, and inflammation within the tumor microenvironment. With the introduction of immunomodulatory drugs and proteasome inhibitors, the prognosis of MM patients has substantially improved. Bortezomib directly inhibits the proliferation of MM cells, induces apoptosis, and affects MM cell interactions with the BM microenvironment by blocking cytokine circuits (76, 77). Although the effectiveness of bortezomib has been demonstrated in MM patients, relapse due to bortezomib-resistance is inevitable and the disease, as it currently stands, remains incurable (78).

**Figure 3:**
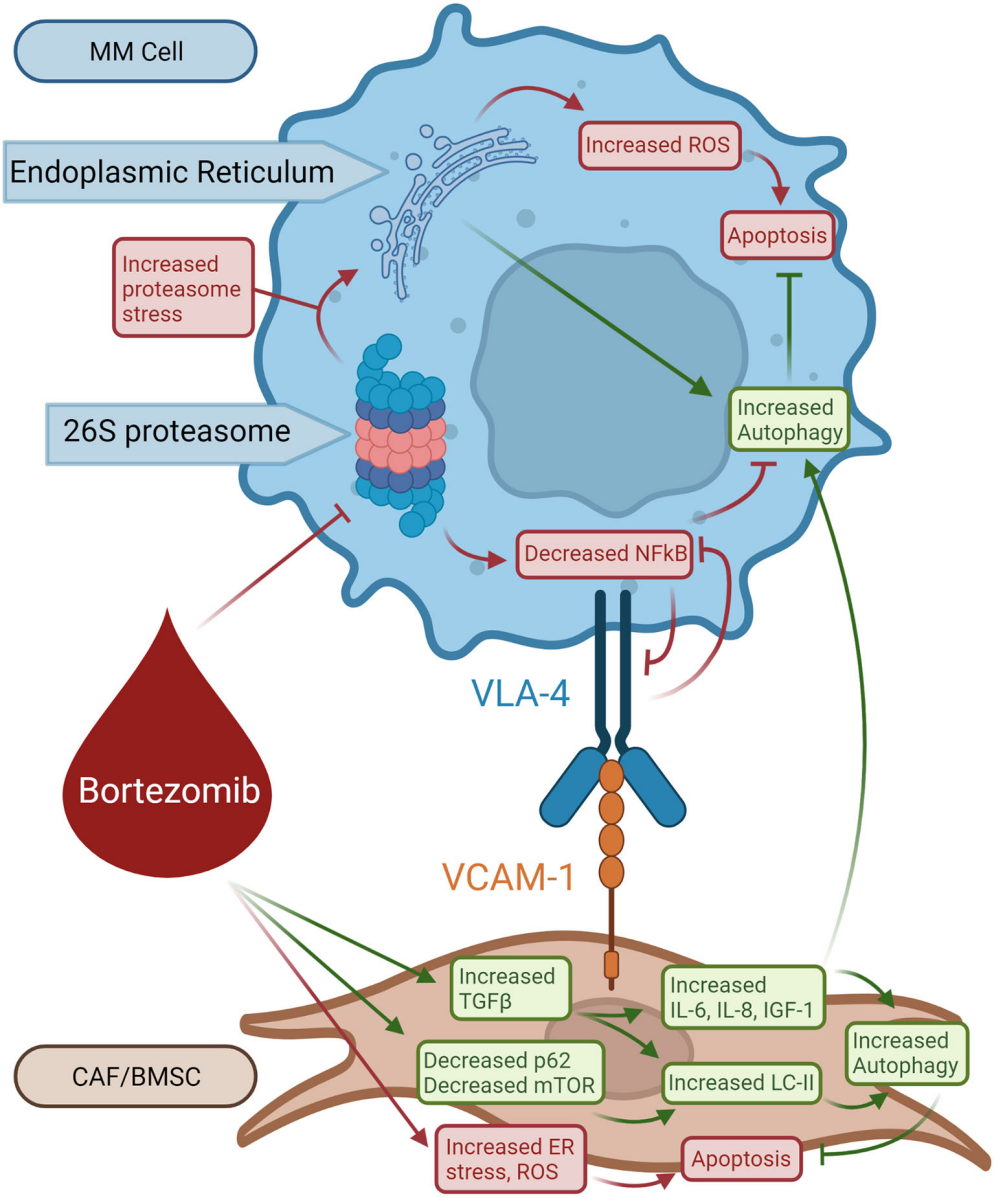
Role of autophagy in bortezomib-induced resistant MM cells. Bortezomib inhibits 26S proteasome activity increasing proteasome stress and decreasing NFkB activity, resulting in decreased VLA-4 expression. Bortezomib also causes ER stress and production of ROS in BMSCs leading to apoptosis. Bortezomib selects for BMSCs that respond with increased TGF-β production. TGF-β increases LC-II, IL-6, IL-8, and IGF1 activity which opposes bortezomib-induced apoptosis and stimulates autophagy in BMSCs. BMSC-produced IL-6, IL-8, and IGF-1 from BMSCs promotes survival in MM cells.

Since bortezomib promotes the accumulation of polyubiquitinated proteins, bortezomib resistance is believed to be mediated by the activation of autophagy (**Figure 3**) (78, 79). The accumulation of proteins induces aggresome and autophagosome formation which can promote protein clearance, tumor survival, and relative drug resistance. Within the BM microenvironment, bortezomib can induce ROS and autophagy through its interaction with cancer-associated fibroblasts (CAFs) through inhibition of mTOR and p62 (78, 79). CAFs are important within the BM stroma and promote cancer initiation, progression, and drug resistance. Co-cultures of MM cells and MM CAFs are resistant to bortezomib *in vitro*, implying that MM CAFs prevent bortezomib-induced apoptosis (80). It was also demonstrated that bortezomib treatment activates autophagy in myeloma CAFs through inhibition of mTOR and p62, induction of LC3, and activation of TGF-β (**Figure 3**) (79). Increased autophagy counteracts proteosome inhibition-induced damage and prevents apoptosis.

This eventual drug resistance is a significant barrier to treatment and ultimate survival in patients with MM. As mentioned before, there is no known cure for MM. As bortezomib resistance develops, researchers have looked for ways to overcome resistance by finding key resistance mechanisms that could be targeted (81). Based on the pathophysiology of bortezomib resistance, autophagy plays a significant role in avoiding apoptosis despite increased stress (78). As previously discussed, caspase 10 is a regulator of autophagy that prevents autophagic cell death (46). If this mechanism for autophagy prosurvival attenuation is disrupted, the autophagy induced by bortezomib therapy and resistance may lead to autophagic cell death. Continued research in the development and use of caspase 10 inhibitors, such as Z-AEVD-FMK, may be beneficial in conjunction with bortezomib to push resistant MM cells towards cell death. More effective treatments may also embrace implementing 3D models based on the patient BM microenvironment to better simulate the patient’s disease progression and test potential treatments (43, 82). These 3D models can also be used to closely monitor progression of the disease for the purpose of rapid modification of treatment (43).

## Discussion

MM is a devastating disease hijacking the delicate balance of antibody production in differentiated PCs. It does so through various mutations and cellular responses that increase activation of the UPR system to increase protein synthesis capacity while simultaneously increasing autophagy to limit proteotoxicity and inhibit apoptosis (83, 84). It also utilizes other signaling systems such as caspase 10 to evade cell death that can result from this abnormal increase in autophagy (41). Autophagy itself has been shown to be involved not only in MM cell survival but also homing to the BM, an important step in disease progression (23, 25). Even when mice deficient in ATG5, a key autophagy protein, were examined, the BM was almost exclusively colonized by ATG5 competent PCs (23, 25). Somewhat paradoxically, given the protein degradation qualities of autophagy, long-term excessive antibody production is associated with an intact autophagic system. Without it, overproduction of Ig from an unchecked UPR system will cause the cells to ultimately produce fewer antibodies (23, 24). The role of the BM microenvironment has also proven to be significant in the development of MM, including its ability to upregulate autophagy (43). The act of PCs, and by extension MM cells, binding to elements of the BM such as BM stromal cells and fibronectin causes pro-survival and proliferative cytokine loops. These signaling loops, driven by NFkB production, release IL-6, TNF-α, and VEGF (43, 59, 63). IL-6 overproduction has been noted in several types of MM and is associated with pathways that may increase UPR and autophagy activity (56). These pro-survival effects from BM binding have also been associated with drug resistance, one of the factors that makes this cancer so deadly. While drugs like Bortezomib attempt to utilize the state of ER stress found in MM cells to drive them towards apoptosis, autophagy is suspected to contribute to resistance and relapse (78, 79). However, as understanding of the roles of autophagy and the BM microenvironment grow, the hope of finding effective treatments and methods of preventing resistance increases.

## Author Contributions

KRH wrote the manuscript and designed **Figures 1**-**3**. KAH contributed to the writing of several subsections of the manuscript. RG provided proofreading and advice given his knowledge of the topic. SA provided mentorship and proofreading and was the PI overseeing the paper. All authors contributed to the article and approved the submitted version.

## Funding

HSC Translational Cancer Research Grant to SA.

## Conflict of Interest

SA, RG, and KAH were authors of one of the articles cited in this paper. The title of said paper is Structural and Ultrastructural Analysis of the Multiple Myeloma Cell Niche and a Patient-Specific Model of Plasma Cell Dysfunction published in the journal Microscopy and Microanalysis. KAH is employed by Organogenesis.

The remaining authors declare that the research was conducted in the absence of any commercial or financial relationships that could be constructed as a potential conflict of interest.

## Publisher’s Note

All claims expressed in this article are solely those of the authors and do not necessarily represent those of their affiliated organizations, or those of the publisher, the editors and the reviewers. Any product that may be evaluated in this article, or claim that may be made by its manufacturer, is not guaranteed or endorsed by the publisher.

## Abbreviations

APRIL, a proliferation-inducing ligand; ATF4, activating transcription factor 4; ATF6, activating transcription factor 6; ATG, autophagy related; BAFF, B cell-activating factor; BCL2, B-cell lymphoma 2; BCMA, B-cell maturation antigen; BiP, immunoglobulin heavy chain-binding protein; BLCAF1, Bcl-2-associated transcription factor 1; Blimp-1, B-lymphocyte-induced maturation protein 1; BM, bone marrow; BMSC, Bone marrow stromal cell; CAF, Cancer-associated fibroblasts; CMA, chaperone-mediated autophagy; ECM, extracellular matrix; eIF2ak3, eukaryotic translation initiation factor 2 alpha kinase 3; eIF2B, eukaryotic translation initiation factor 2B; ER, endoplasmic reticulum; ERAD, ER associated degradation; GRP78, glucose regulated protein 78; HIF1a, Hypoxia-inducible factor 1-alpha; ICAM-1, Intracellular adhesion molecule 1; Ig, immunoglobulin; IL-6, interleukin 6; IRE1, inositol-requiring enzyme 1; IRF4, interferon regulatory factor 4; LAMP-2A, lysosomal-associated membrane protein 2A; LC3, Microtubule associated protein light chain 3; LFA-1, Lymphocyte function-associated antigen 1; MGUS, monoclonal gammopathy of undetermined significance; MM, multiple myeloma; mTOR, mammalian target of rapamycin; mTORC1, mammalian target of rapamycin complex 1; NFkB, nuclear factor kappa B; NOS, nitrous oxide; NRF2, Nuclear factor erythroid 2–related factor 2; PC, plasma cell; PERK, PKR-like endoplasmic reticulum kinase; Rag2, Recombination activating 2; RIDD, regulated IRE1-dependent decay; ROS, reactive oxygen species; SQSTM1, sequestosome 1; TACI, transmembrane activator and CAML interactor; TNF-α, Tumor necrosis factor alpha; TRAF, tumor necrosis factor receptor-associated factors; UPR, unfolded protein response; UPS, ubiquitin proteosome system; VEGF, Vascular endothelial growth factor; VLA-4, very late antigen 4; XBP-1, X-Box Binding Protein 1.

## Glossary

**Table:**

APRIL	a proliferation-inducing ligand
ATF4	activating transcription factor 4
ATF6	activating transcription factor 6
ATG	autophagy related
BAFF	B cell-activating factor
BCL2	B-cell lymphoma 2
BCMA	B-cell maturation antigen
BiP	immunoglobulin heavy chain-binding protein
BLCAF1	Bcl-2-associated transcription factor 1
Blimp-1	B-lymphocyte-induced maturation protein 1
BM	bone marrow
BMSC	Bone marrow stromal cell
CAF	Cancer-associated fibroblasts
CMA	chaperone-mediated autophagy
ECM	extracellular matrix
eIF2ak3	eukaryotic translation initiation factor 2 alpha kinase 3
eIF2B	eukaryotic translation initiation factor 2B
ER	endoplasmic reticulum
ERAD	ER associated degradation
GRP78	glucose regulated protein 78
HIF1a	Hypoxia-inducible factor 1-alpha
ICAM-1	Intracellular adhesion molecule 1
Ig	immunoglobulin
IL-6	interleukin 6
IRE1	inositol-requiring enzyme 1
IRF4	interferon regulatory factor 4
LAMP-2A	lysosomal-associated membrane protein 2A
LC3	Microtubule associated protein light chain 3
LFA-1	Lymphocyte function-associated antigen 1
MGUS	monoclonal gammopathy of undetermined significance
MM	multiple myeloma
Mtor	mammalian target of rapamycin
mTORC1	mammalian target of rapamycin complex 1
NFkB	nuclear factor kappa B
NOS	nitrous oxide
NRF2	Nuclear factor erythroid 2–related factor 2
PC	plasma cell
PERK	PKR-like endoplasmic reticulum kinase
Rag2	Recombination activating 2
RIDD	regulated IRE1-dependent decay
ROS	reactive oxygen species
SQSTM1	sequestosome 1
TACI	transmembrane activator and CAML interactor
TNF-a	Tumor necrosis factor alpha
TRAF	tumor necrosis factor receptor-associated factors
UPR	unfolded protein response
UPS	ubiquitin proteosome system
VEGF	Vascular endothelial growth factor
VLA-4	very late antigen 4
XBP-1	X-Box Binding Protein 1
